# Analysis of the Rolling Interface Contact Characteristics in Mixed Lubrication Based on Gaussian Distribution Theory

**DOI:** 10.3390/ma16155220

**Published:** 2023-07-25

**Authors:** Laihua Tao, Qiaoyi Wang, Ziwei Qi, Huajie Wu, Hanbin Zhu, Junbo Huang

**Affiliations:** 1 School of Mechanical Engineering, Hangzhou Dianzi University, Hangzhou 310018, China; taolh@zjweu.edu.cn (L.T.); whj_china@163.com (H.W.); 18720104694@163.com (H.Z.); 13789115352@163.com (J.H.); 2 School of Mechanical and Automotive Engineering, Zhejiang University of Water Resources and Electric Power, Hangzhou 310018, China

**Keywords:** rolling interface, Gaussian rough surface, mixed lubrication, fluid–structure interaction (FSI), contact characteristics

## Abstract

To reveal the influence of surface morphology characteristics in mixed lubrication on the contact characteristics of the rolling interface, a random three-dimensional rough surface model based on Gaussian distribution theory was established. The model utilizes the finite element method (FEM) to simulate the regular contact and tangential sliding behavior of micro-asperities at the rolling interface in mixed lubrication conditions. The connection bearing capacity of models with varied roughness in mixed lubrication was studied. Furthermore, the effect of various sliding and normal indentation amounts on the normal and friction stress was investigated. The simulation result reveals that the roughness of the surface influences the distribution of the lubricating oil film. The lubricating oil layer between the interfaces with a lower roughness has a higher bearing capacity due to its more uniform distribution of peaks and valleys. An increase in the normal indentation amount raises the friction stress and normal stress. In contrast, an increase in sliding lowers the normal pressure, substantially impacting the fluctuation of the friction coefficient dramatically. Finally, the random three-dimensional rough surface model is verified by comparing it with the experimental data in the related literature.

## 1. Introduction

In the research field of rolling, the rolling interface is rough at the microscopic scale [1], and solid–solid contact between the roller and the strip occurs at the peak of the roughness. During the metal-strip-rolling process, the peaks and valleys on the contact surface typically bear most of the interface load, causing higher-hardness asperities to undergo elastic deformation and then transfer the contour onto the metal strip, resulting in plastic deformation of its surface. This deformation will have a significant impact on the friction and lubrication condition at the rolling interface, leading to changes in the morphology of the metal strip and, consequently, reducing the rolling performance and surface smoothness of the strip. The interface friction and contact during metal rolling have been extensively studied by scholars; it is of great significance to study and optimize the friction and lubrication properties of the rolling interface to achieve the high-quality rolling of the plate and strip.

In terms of the friction and lubrication of solid contact at the rolling interface, Wu et al. [2,3] presented a multiscale soft-contact modelling (SCM) method for characterizing the rough surfaces in contact with coupled slipping/sliding and rolling, to analyze the interaction between two contact bodies at the interface under sliding and rolling coupling conditions. However, no simulation was conducted on the tangential slip behavior of the micro-asperities at the rolling interface. The key factors affecting the rolling quality of metal strips include fluid lubrication at the rolling interface, random asperity contact, and asperity–lubricant interaction [4]. Using a mixed lubrication model, Zhang et al. [5] proposed a boundary film strength model that can comprehensively reflect the influences of film thickness, pressure, and shear stress; a mixed lubrication model considering boundary film strength was established by coupling the boundary film strength model with the hydrodynamic lubrication model and the asperity contact model. However, the accuracy of the asperity contact model needs further in-depth research. Jeng et al. [6] examined the effects of the roll speed, reduction rate and lubricant viscosity on the contact ratio, and roll pressure of cold-rolled aluminum sheets; the numerical analysis of the contact ratio is conducted under nominal operating conditions. Xia et al. [7] investigated the micro-scale behavior of a single contact point on the surface of a working roll in mixed lubrication conditions using three-dimensional FEM. However, the model did not predict the actual working conditions of the rolling interface very well. Chen et al. [8] studied the impact of roller surface roughness on surface quality, surface micro-defects. and rolling force during the cold rolling process of stainless steel through cold rolling lubrication experiments. However, the mechanism of the influence of surface roughness on lubrication friction needs further research. According to the evolution law of surface morphology in the rolling process, Zhang and Sutcliffe et al. [9,10] formed a generative model of surface morphology in the rolling process integrating rough contact, rolling force, and relative slip between roll and strip, which obtained the change rules of the strip surface morphology with the influence of process parameters. Despite considering the effect of the rough surface morphology on contact characteristics during the rolling process, their study on the real rough surfaces of rolls and rolled parts during the rolling process was restricted, reducing the dependability of models.

The roller and workpiece’s working surface is a random rough surface during the rolling operation. Hu and Wu et al. [11,12] studied the influence of different shape textures at the working interface on tribological properties, and also analyzed the relationship between various textures, micro-roughness, macro roll deformation and friction stress, and hydrodynamic lubrication, but did not study in depth the influence mechanism of micro-scale contact surface and friction lubrication. In recent years, much research has been carried out on the influence mechanism of rolling interface roughness and mixed lubrication. Shi et al. [13] developed a micro-rough surface contact calculation model and examined the influence of load on elastic–plastic deformation using FEM. Zhao et al. [14] established a numerical model for predicting surface contact characteristics during surface wear processes based on non-Gaussian surface reconstruction technology and sliding wear models to study the evolution of the morphology of Gaussian rough surfaces during wear processes. Megalingam et al. [15] used FEM to carry out three-dimensional contact analyses of Gaussian rough surfaces to determine the effect of surface arithmetic mean height on the contact pair. Thirumalai et al. [16] conducted several experiments by varying the cutting speed, feed, and depth of cut as machining parameters based on the design of experiments, and the surface roughness and flank wear are measured as responses against these parameters. Li et al. [17] obtained the modeling approach of the quadratic function of revolving-body-equivalent micro-asperity by the rough surface topography measurement experiment, which increases the accuracy of the contact characteristic analysis of joint surfaces. However, existing research has not combined the three-dimensional random surface generation technology with lubricants in the rolling work’s deformation zone. This leads to a lack of research on the micro-scale rough contact and the interaction between roughness and lubricants at the rolling interface. Under the participation of lubricants, various parts of the roller bite will undergo fluid dynamic pressure lubrication, mixed lubrication, and boundary lubrication [18,19], forming a coupled solid–solid (rough–rough) and solid–liquid (rough–lubricant) contact [20]. Therefore, it is necessary to consider and investigate the rough contact between randomly rough surfaces at the micro scale of the rolling interface, and the impact of lubricant.

In conclusion, this paper investigates the frictional contact process of rough surfaces with a Gaussian distribution in a mixed lubrication condition during rolling to reveal the rough contact characteristics at the micro level under lubrication. In comparison to Christensen’s approximate value [21], which is used by most researchers to replace the Gaussian distribution, the three-dimensional random surface generation technique employed in the article has improved the accuracy of the actual distribution of the comprehensive roughness of the roller and strip surfaces, which enhanced the precision of the established calculation model.

## 2. Micro-Scale Simulation Model

### 2.1. Gaussian Control Equation for Rough Surfaces

**Figure 1:**
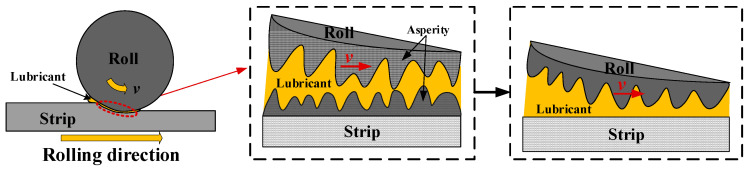
Microscopic equivalent model of contact between the roll and roll strip.

When the rollers come into contact with the strip, the rough peaks on the rollers typically undergo elastic deformation; in contrast, the rough peaks on the strip undergo elastic–plastic deformation because of the significant differences in hardness between the rollers and the strip. The maximum pressure at which the rollers and strip come into contact depends on the yield strength of the strip. The contact between two rough surfaces can be equivalently treated as the contact between a rough surface and a smooth surface, as shown in Figure 1.

The composited roughness $\sigma_{m}$ is given by

(1) $$\sigma_{m}=\sqrt{\sigma_{s}^{2}+\sigma_{r}^{2}}$$

where $\sigma_{r}$ and $\sigma_{s}$ are the surface roughness of the roll and strip, respectively. Because of the random distribution of the surface asperity heights, Mccool [22] provided the standard deviation for the highly random distribution of surface roughness, i.e., the root-mean-square (RMS) roughness $\sigma_{a}$ of the surface.

(2) $$\sigma_{a}=\sigma_{m}\sqrt{1-\frac{3.717\times 10^{-4}}{{\left(N_{a}R_{a}\sigma_{m}\right)}^{2}}}$$

where $N_{a}$ and $R_{a}$ are the asperity density and average asperity radius.

Gaussian distribution of asperity heights was represented as $f\left(\delta \right)$

(3) $$f\left(\delta \right)=\frac{\exp(-\frac{1}{2}{\left(\frac{\delta }{\sigma_{a}}\right)}^{2})}{\sigma_{a}\sqrt{2\pi }}$$

where δ is measured concerning the mean plane of asperity heights.

### 2.2. Three-Dimensional Surface Modeling and Analysis of Gaussian-Distributed Rough Surfaces

Extensive experimental research has found that the height distribution of micro-asperities between the rough surface of the work roll and strip conforms to the Gaussian distribution law. According to the three-dimensional simulation method for rough surfaces based on the autocorrelation function proposed by Chen [23,24], the exponential autocorrelation function $R\left(\tau_{x},\tau_{y}\right)$ can be expressed as:

(4) $$R\left(\tau_{x},\tau_{y}\right)=\sigma_{a}^{2}\exp\left[-2.3{\left({\left(\frac{\tau_{x}}{\beta_{x}}\right)}^{2}+{\left(\frac{\tau_{y}}{\beta_{y}}\right)}^{2}\right)}^{\frac{1}{2}}\right]$$

where $\beta_{x}$ and $\beta_{y}$ are the correlated lengths in the x and y directions, respectively.

**Figure 2:**
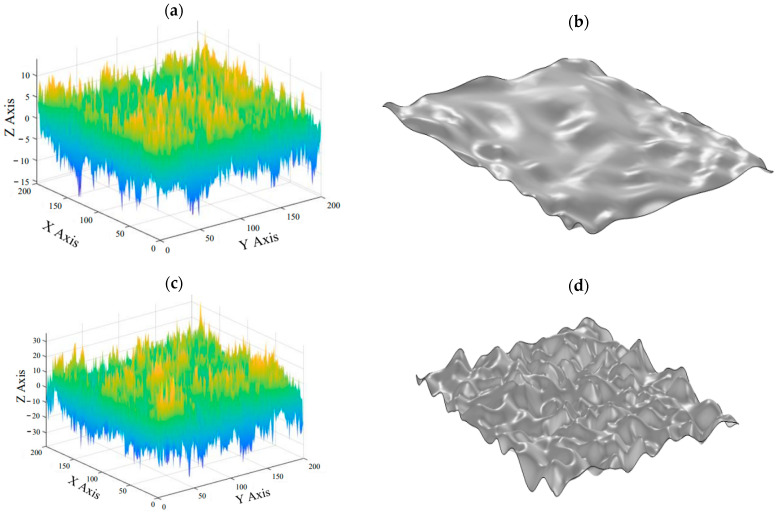
A three-dimensional Gaussian rough surface point cloud map and parameterized surface model with varying RMS values: (**a**,**b**) the Gaussian rough surface point cloud map and parameterized surface model with σ_1 = 2 μm; AND (**c**,**d**) the Gaussian rough surface point cloud map and parameterized surface model with σ_2 = 5 μm.

This article generates isotropic Gaussian rough surface point cloud files with surface RMS roughness values of $\sigma_{1}=2\;\mu m$ and $\sigma_{2}=5\;\mu m$, and autocorrelation length $\beta_{x}=\beta_{y}=100$, by using MATLAB. The files are imported into three-dimensional modeling software for interpolating fitting and parameterized surface modeling, as shown in Figure 2.

### 2.3. Establishment and Analysis of Calculation Model for Rolling Interface

**Figure 3:**
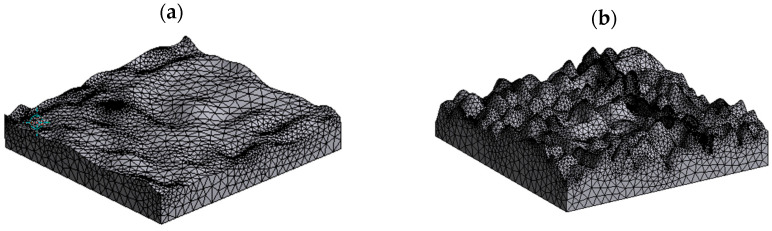
Calculation model of the roller rough surface with varying RMS values: (**a**) $\sigma_{1}=2\;\mu m$; and (**b**) $\sigma_{2}=5\;\mu m$.

To ensure the computational efficiency and accuracy of the model, the contact between two rough surfaces is equivalently treated as the contact between a rough surface and a smooth surface. The micro-surface of the roller is taken as the rough surface. As shown in Figure 3, the three-dimensional model of the rough surfaces with different RMS values of $\sigma_{1}=2\;\mu m$ and $\sigma_{2}=5\;\mu m$ are created.

**Figure 4:**
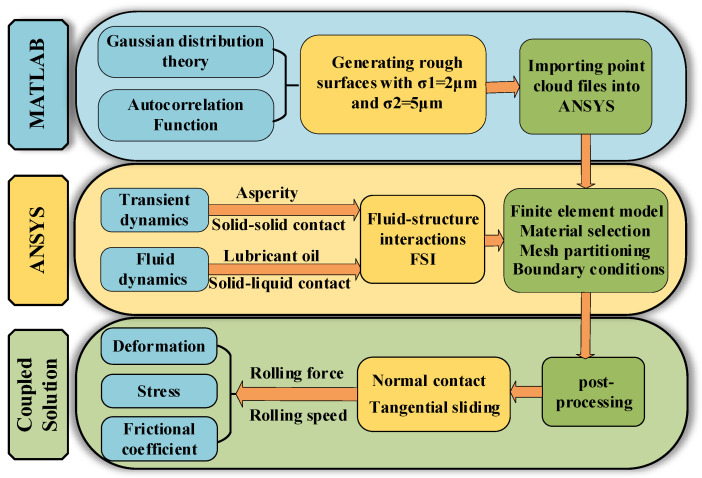
Process flowchart for establishment and analysis of rolling interface calculation model.

To simulate the effects of rolling conditions on the contact characteristics of the rolling interface under different roughness levels, three-dimensional models of rough surfaces with $\sigma_{1}=2\;\mu m$ and $\sigma_{2}=5\;\mu m$ are imported into FEM software version 19.0, then modeled, simulated, and analyzed according to the process shown in Figure 4.

**Figure 5:**
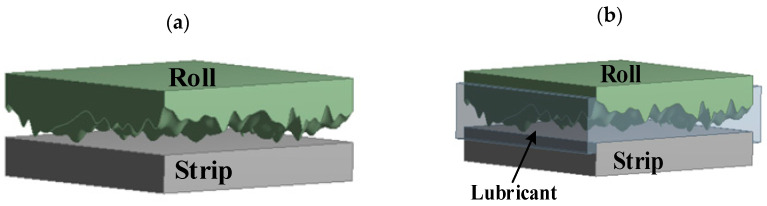
Calculation model of the rolling interface in different rolling conditions: (**a**) model in dry friction; and (**b**) model in mixed lubrication.

The condition of the deformation zone is very complex during the rolling process. In past research, the involvement of lubricants in the rolling process was frequently overlooked to simplify the analysis model, and the rolling process was simplified as rough surfaces contacting each other in dry friction. In this state, the contact of the working interface is a solid–solid contact, and the calculation model consists of two solid domains, which requires transient dynamics for simulation calculation, as shown in Figure 5a. However, when the rolling interface is in mixed lubrication, the rolling roller draws lubricant into the rolling interface’s work zone. This results in the partial sharing of rolling pressure by fluid dynamic pressure and the contact of micro-asperities. Therefore, the importance of lubricants in the rolling process cannot be ignored. At this moment, the rolling interface contact is a coupled contact of solid–solid and fluid–solid. Therefore, the calculation model consists of two solid domains and one fluid domain, and requires combined transient dynamics and fluid dynamics for FSI simulation, as shown in Figure 5b.

## 3. Simulation Process

### 3.1. Performance Parameters of Materials

Considering the high yield strength of the work rolls, carbon steel is adopted for the rolling rolls, aluminum alloy for the strips, and lubricant and lubricant is chosen as the engineering equipment lubricating oil. The specific parameters are shown in Table 1.

### 3.2. Boundary Conditions of Dry Friction Model

**Figure 6:**
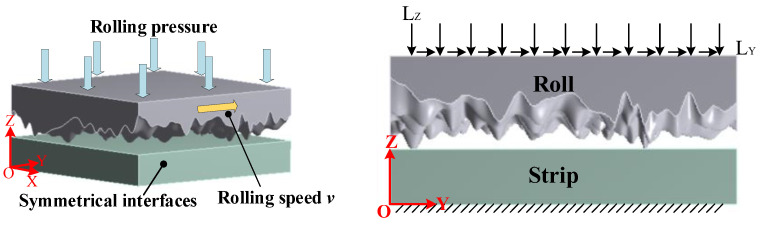
Boundary condition at the rolling interface in dry friction.

When the rolling interface is in dry friction, the rolling pressure is carried by the contact of the micro-asperities. Figure 6 depicts the boundary conditions applied in the rolling work zone under dry friction conditions. The Y-axis direction is defined as the rolling direction. The rolling pressure can be equivalently treated as the displacement load $L_{Z}$ along the negative direction of the Z-axis due to the linear relationship between the rolling pressure and the pressed displacement, while, at the microscopic scale, rolling speed is considered to be horizontal in the rolling work zone, so it can be represented as a displacement load $L_{Y}$ along the rolling direction in unit time. Thus, the application of specific boundary conditions is divided into two steps: The first step is to apply a normal displacement load $L_{Z}$ on the rough surface of the roll, causing the roll to press against the strip, and the rough peaks on the roll surface will contact the strip, which leads to the forming of indentations. In the second step, a tangential displacement load $L_{Y}$ is added along the rolling direction based on the first step to induce sliding between the rolling mill and the strip. Considering the lateral flow of metal plates during the rolling process, symmetrical interfaces are established on two faces perpendicular to the rolling direction of the plate to prevent lateral metal flow. Moreover, the bottom face of the plate is set as the fixed surface.

### 3.3. Boundary Conditions of Mixed Lubrication Model

**Figure 7:**
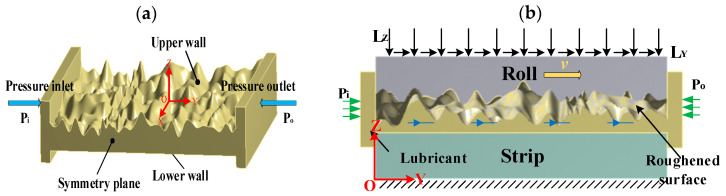
Boundary condition at the rolling interface in mixed lubrication: (**a**) fluid boundary condition; and (**b**) boundary condition of FSI model.

When the rolling interface is in mixed lubrication, the lubricant is sucked into the work zone of the rolling interface by the rolling roller, causing a partial sharing of the rolling pressure by the fluid dynamic pressure and the contact of the micro-asperities. Figure 7a,b show a schematic diagram of the boundary conditions applied in the working zone of the rolling process under mixed lubrication conditions. Compared to the dry friction model, the mixed lubrication model increases the effect of lubricant in the rolling work zone, therefore, it is necessary to establish a fluid dynamics model to analyze the mechanical properties of the lubricant. Because of the high lubrication pressure, the lubricant is generally treated as a Newtonian fluid with incompressibility in computational fluid dynamics (CFD), and the lubricant flow follows the laminar model, which can be calculated using the Navier–Stokes equation.

(5) $$\rho \frac{\partial v}{\partial t}=\eta \nabla^{2}v-\nabla p-\frac{\eta }{3}\nabla \left(\nabla \cdot v\right)+\rho f$$

where ρ is the lubricant density, p is the compressive pressure, η is the dynamic viscosity, v is the flow velocity, f is other external forces, and ∇ is the divergence operator. Lubricants are continuous in the flow process and follow the conservation principles of mass, momentum, and energy.

Lubricants are continuous during the flow process and adhere to mass, momentum, and energy conservation laws. Based on the continuity equation, the conservation of mass may be represented as follows:

(6) $$\frac{\partial \rho }{\partial t}+\nabla \cdot \left(\rho v\right)=0$$

The momentum conservation equation can be expressed as:

(7) $$\frac{\partial v}{\partial t}+\left(v\cdot \nabla \right)v=\frac{1}{\rho }\nabla \cdot \sigma +f$$

The energy conservation equation can be expressed as:

(8) $$\rho \frac{\partial E_{w}}{\partial t}=\left(p-p_{v}\right)\frac{1}{\rho }\frac{\partial \rho }{\partial t}+S:\sigma$$

where $E_{w}$ represents the internal energy, $p_{v}$ is the pressure caused by fluid viscosity, S is the deviatoric stress tensor, σ is the strain rate deviation, and S:σ is the double dot product of tensors.

In the calculation model of fluid, the input pressure $P_{i}$ and outlet pressure $P_{o}$ are established, and the pressure difference between the inlet and outlet obtains the initial high-pressure fluid dynamics. According to Equations (5)–(8), the fluid flow velocity and pressure on the upper and lower wall surfaces are calculated and imported into the solid domain to analyze the fluid–structure interaction. The boundary conditions for the roller and strip in the solid domain are set in the same way as the dry friction model to obtain the mixed lubrication model. Finally, the lubricant fluid and solid domains formed by the roll strip are coupled for analysis to obtain the plastic deformation, pressure distribution, friction force distribution, and contact bearing capacity of micro-asperities at the rolling interface in mixed lubrication.

## 4. Result Analysis

The established calculation model will be submitted to simulation calculations with the results analyzed in post-processing. The essay will examine the models under two different working conditions: normal contact and tangential sliding.

### 4.1. Normal Contact

**Figure 8:**
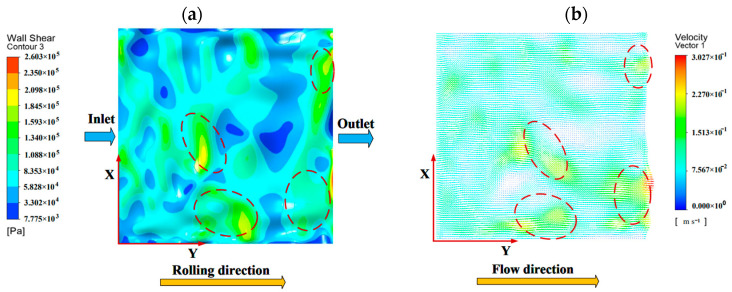

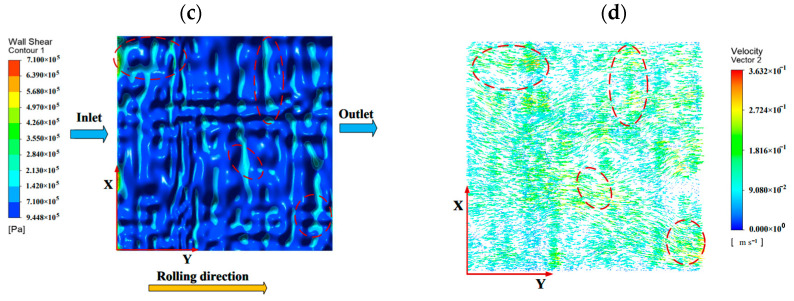
Distribution map of oil film shear stress and velocity under different roughness: (**a**) shear stress with σ_1 = 2 μm; (**b**) velocity with σ_1 = 2 μm; (**c**) shear stress with σ_2 = 5 μm; and (**d**) velocity with σ_2 = 5 μm.

**Figure 9:**
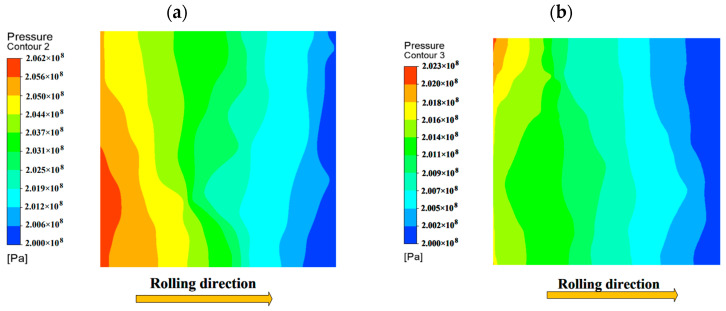
Distribution map of oil film pressure under different roughness: (**a**) σ_1 = 2 μm; and (**b**) σ_2 = 5 μm.

Distribution of oil film pressure and velocity on rough surfaces. The relationship between oil film shear stress and speed under different roughness is shown in Figure 8. From Figure 8a,b, it can be seen that the oil film velocity is higher and the shear stress is relatively larger at the troughs of rough surfaces. Moreover, the unevenness of the rough surface will seriously affect the distribution of oil film stress, as shown in Figure 8a,c. When the roughness is $\sigma_{1}=2\;\mu m$, the stress and velocity distribution inside the oil film are relatively uniform. However, when the roughness is $\sigma_{2}=5\;\mu m$, the oil film cannot fill the entire peak during the flow process due to the large fluctuation amplitude of the rough surface peaks and troughs, resulting in unstable stress distribution and velocity on the rough surface. Therefore, the overall normal load-carrying capacity of the oil film is relatively weak at $\sigma_{2}=5\;\mu m$. As shown in Figure 9, the average normal load-carrying capacity is greater when $\sigma_{1}=2\;\mu m$ than $\sigma_{2}=5\;\mu m$. The initial oil film pressure exhibits a step-like distribution along the rolling direction. This is because the rough surface peaks and valleys change the fluid velocity inside, causing pressure loss during fluid flow along the rolling direction, and resulting in a step-like oil film pressure distribution.

**Figure 10:**
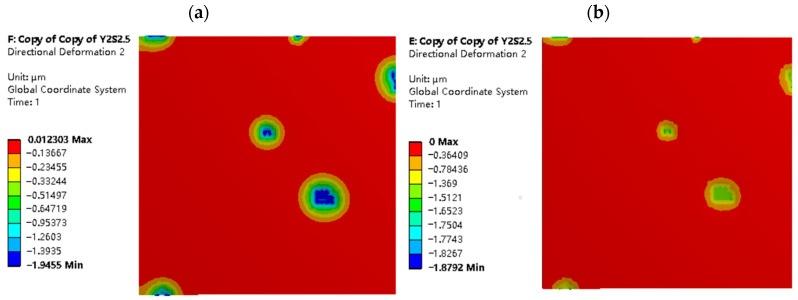
Normal deformation map of strip with σ_1 = 2 μm and L_Z = 2 μm: (**a**) dry friction model; and (**b**) mixed lubrication model.

**Figure 11:**
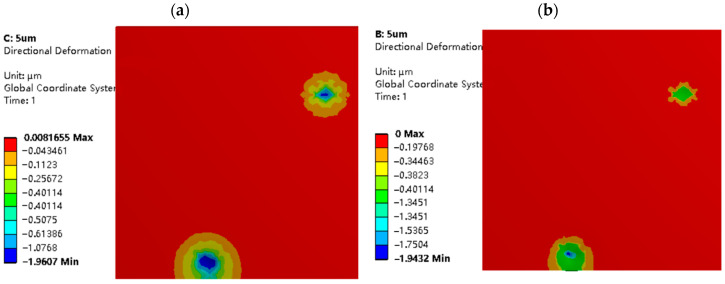
Normal deformation map of strip in different rolling conditions with σ_2 = 5 μm and L_Z = 2 μm: (**a**) model in dry friction; and (**b**) model in mixed lubrication.

Normal plastic deformation of the strip. Figure 10 and Figure 11 illustrate the distribution of normal deformation on the strip surface under the same indentation depth for different working conditions and roughness models. The simulation results indicate that the number and area of deformation regions on the strip surface are higher for $\sigma_{1}=2\;\mu m$ than for $\sigma_{2}=5\;\mu m$, when the normal displacement load $L_{Z}=2\;\mu m$ is applied to both roughness models. This is because the RMS value of the working surface increases with the roughness, resulting in larger peak-to-valley amplitudes of the asperities and higher surface non-uniformity during rolling. The surface non-uniformity of the rough surface with $\sigma_{2}=5\;\mu m$ is higher than that of the surface with $\sigma_{1}=2\;\mu m$, thus resulting in a smaller number of contact peaks on the strip surface for $\sigma_{2}=5\;\mu m$ when the same normal displacement load $L_{Z}$ is applied.

From Figure 10 and Figure 11, it is evident that the maximum normal deformation and area of the mixed lubrication model with $\sigma_{1}=2\;\mu m$ and $\sigma_{2}=5\;\mu m$ are smaller than those of the dry friction model. This phenomenon can be attributed to the involvement of lubricant, which helps to distribute the rolling pressure between fluid dynamic pressure and solid contact pressure, and balances the interface load distribution. Additionally, when the normal displacement load $L_{Z}$ is the same, the larger the roughness, the greater the amplitude of the surface roughness peaks and valleys, and the fewer the contact peaks at the rolling interface. As a result, the rolling pressure borne by each individual peak increases, so the maximum deformation of $\sigma_{2}=5\;\mu m$ is always greater than that of $\sigma_{1}=2\;\mu m$.

**Figure 12:**
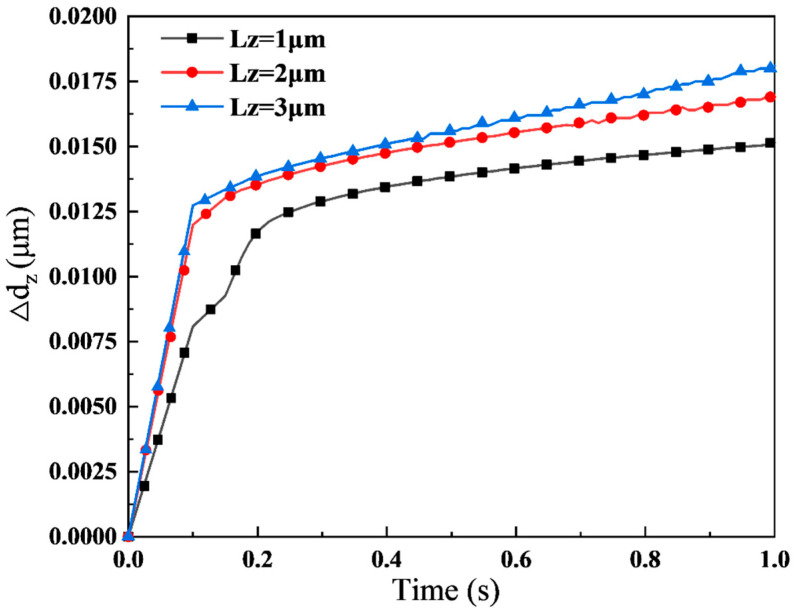
Time-varying curve of ∆d_Z under different normal displacement loads L_Z when σ_2 = 5 μm.

Figure 12 shows the time-dependent variation of $∆d_{Z}$ under different normal displacement loads $L_{Z}$ when $\sigma_{2}=5\;\mu m$. To characterize the computational error induced by the neglect of lubricant effects in the model, $∆d_{Z}$ is defined as the disparity in normal deformation between two working conditions.

(9) $$∆d_{Z}=d_{dry}-d_{mix}$$

where $d_{dry}$ is the normal deformation of the strip in dry friction, and $d_{mix}$ represents the normal deformation of the strip in mixed lubrication.

The simulation results demonstrate that $∆d_{Z}$ increases with the augmentation of the normal displacement load. The greater the normal indentation, the more significant the difference in normal deformation $∆d_{Z}$, as the lubricant shares a higher rolling pressure. Therefore, the role of lubricant cannot be ignored in the rolling process.

**Figure 13:**
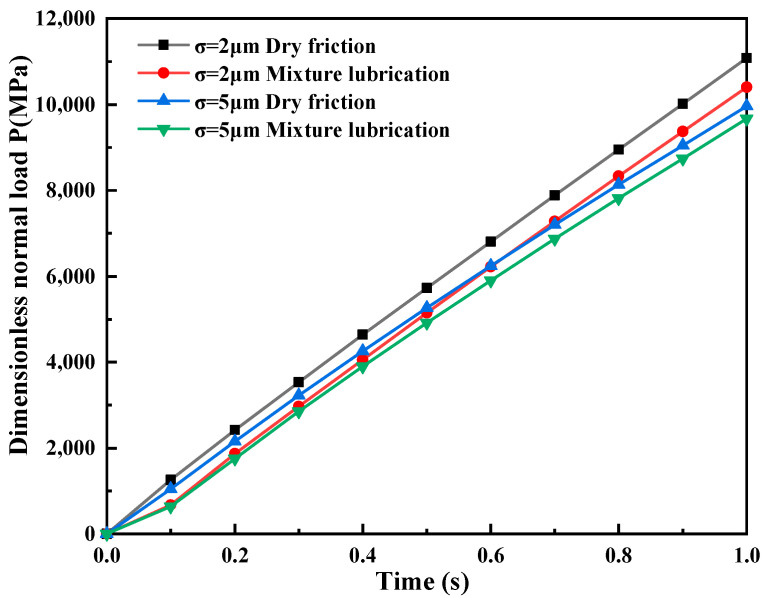
The variation of dimensionless normal load P with time for σ_1 = 2 μm and σ_2 = 5 μm.

Normal Contact Bearing Capacity. Figure 13 shows the temporal variation of dimensionless normal load P for different working conditions and roughness models at the same indentation depth. The normal load F is obtained through post-processing, and, combined with the nominal contact area *S*_0_ = 200 μm × 200 μm, P is calculated as

(10) $$P=\frac{F}{S_{0}}$$

The simulation result reveals that the micro-asperities exhibit a higher normal load-carrying capacity for $\sigma_{1}=2\;\mu m$ than for $\sigma_{2}=5\;\mu m$ in mixed lubrication and dry friction. Moreover, when the indentation depth is constant, the normal load-carrying capacity of the micro-asperities in mixed lubrication is consistently lower than that in dry friction, and the difference in the normal load carried by the micro-asperities between the two working conditions increases with decreasing roughness. This phenomenon is primarily attributed to the fact that the rolling pressure is entirely borne by the micro-asperities in contact with dry friction. In contrast, in mixed lubrication, the lubricant balances the distribution of the rolling interface load. As the roughness decreases, the oil film peak height becomes more uniform, and the pressure loss caused by the unstable flow velocity decreases, resulting in a stronger normal load-carrying capacity of the lubricant. Therefore, the effect of lubrication is more pronounced for $\sigma_{1}=2\;\mu m$.

**Figure 14:**
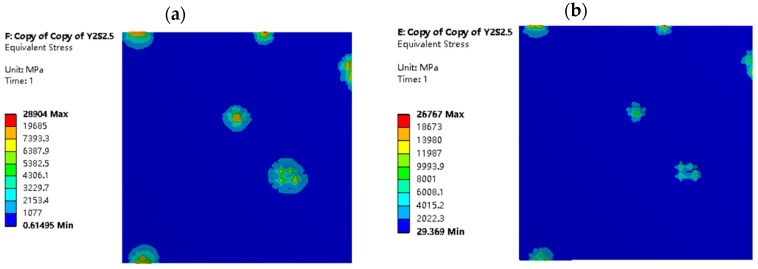
Equivalent stress distribution diagrams of strip in different rolling conditions with σ_1 = 2 μm and L_Z = 2 μm: (**a**) model in dry friction; and (**b**) model in mixed lubrication.

**Figure 15:**
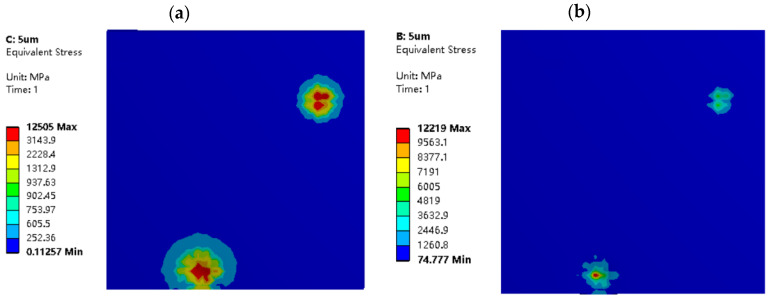
Equivalent stress distribution diagrams of strip in different rolling conditions with σ_2 = 5 μm and L_Z = 2 μm: (**a**) model in dry friction; and (**b**) model in mixed lubrication.

Figure 14 and Figure 15 depict the equivalent stress distribution maps of the dry friction and mixed lubrication model, respectively, at the same indentation depth for different roughness models. When $\sigma_{2}=5\;\mu m$, the stress distribution of the two working conditions is relatively consistent, and the size and number of stress areas are similar. However, when $\sigma_{1}=2\;\mu m$, there are differences in the size and number of stress distribution areas between the two working conditions. This is because, at the same indentation depth ($L_{Z}=2\;\mu m$) and $\sigma_{1}=2\;\mu m$, the distribution of oil film peaks and valleys is more uniform, resulting in a relatively stable internal flow field, higher normal load-carrying capacity, and more pronounced oil film effects. This finding is consistent with the conclusions of Figure 9 and Figure 13, which confirms the model’s reliability.

**Figure 16:**
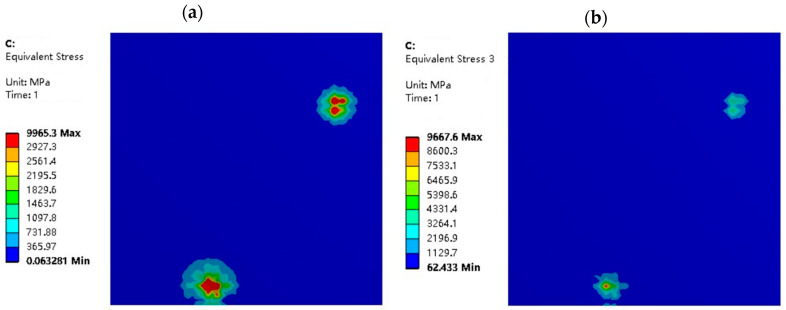
Equivalent stress distribution diagrams of strip in different rolling conditions with $\sigma_{2}=5\;\mu m$ and $L_{Z}=1\;\mu m$: (**a**) model in dry friction; and (**b**) model in mixed lubrication.

Additionally, when the roughness is constant, the amplitude and size of high-stress zones will grow as the usual displacement load increases, as shown in Figure 15 and Figure 16. When $\sigma_{2}=5\;\mu m$, the amplitude and area of high-stress areas with normal displacement $L_{Z}=2\;\mu m$ are significantly larger than those with $L_{Z}=1\;\mu m$. Therefore, when the normal displacement load increases, the degree of extrusion of the roller micro-asperities on the strip surface increases, as does the rolling pressure sustained by the contact micro-asperities.

### 4.2. Tangential Sliding

**Figure 17:**
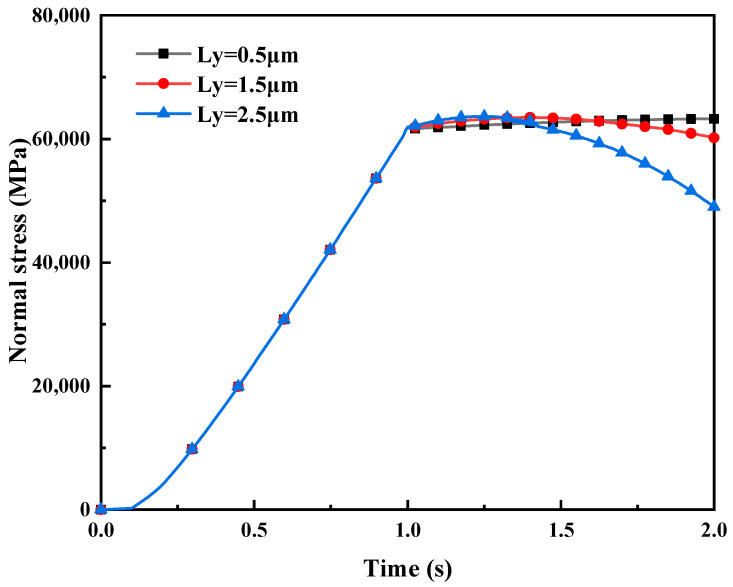
The impact of different tangential displacement load L_Y on normal stress in mixed lubrication with σ_2 = 5 μm.

Normal stress. Figure 17 illustrates the impact of different tangential displacement loads $L_{Y}$ on normal stress in mixed lubrication. Due to the linear relationship between rolling pressure and normal displacement load, the rolling pressure can be represented by the normal displacement load $L_{Z}$, and the rolling speed can be represented by the tangential displacement load $L_{Y}$ along the rolling direction in unit time. To simulate the rolling process, the input of rolling pressure is simulated in the first timestep (t=0~1 s), followed by the input of rolling speed in the second timestep (t=1~2 s).

The simulation results demonstrate that, when the amount of compression ($L_{Z}=1\;\mu m$) remains constant (i.e., the initial rolling pressure input is fixed), an increase in tangential displacement load $L_{Y}$ per unit time leads to a non-linear decrease in normal stress, because, under constant rolling pressure, the real contact area remains roughly stable, and the stress distribution of the contact is relatively uniform. However, with sliding time accumulation from 1 s to 2 s, adhesion and plowing effects cause the elastic–plastic deformation of micro-asperities in contact. Moreover, the micro-asperities will compress the surface of the strip during sliding to form furrows, resulting in an increase in contact area and a decrease in contact stress. Additionally, the larger the tangential displacement load per unit time, i.e., the rolling speed, the faster the lubricant is entrained, and the higher the bearing capacity of the lubricating oil film. The solid contact stress between micro-asperities is further relieved, so the larger the rolling speed, the greater the magnitude of the reduction in contact stress between micro-asperities.

**Figure 18:**
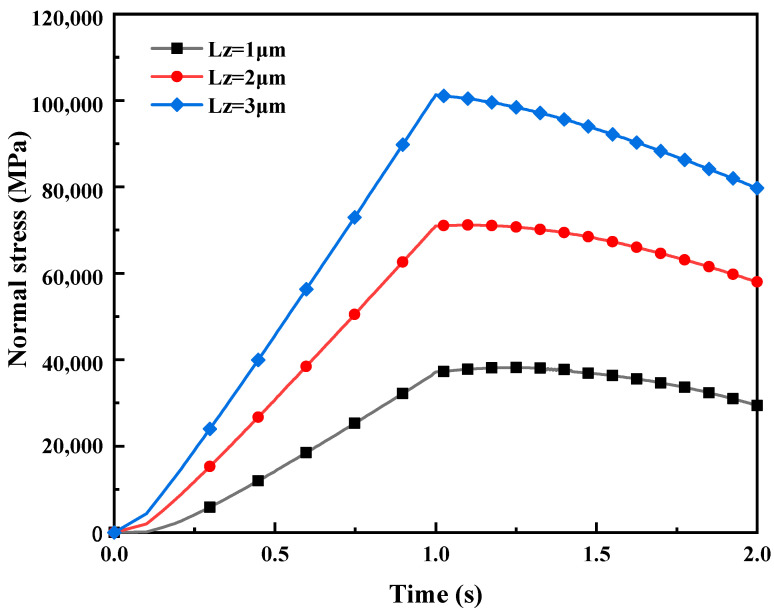
The impact of different displacement loads L_Z on normal stress in mixed lubrication with σ_2 = 5 μm.

Figure 18 depicts the influence of different normal displacement loads $L_{Z}$ on the normal stress in mixed lubrication. Simulation results indicate that, when the tangential displacement load $L_{Y}$ per unit time is constant ($L_{Y}=2.5\;\mu m$) (i.e., the input rolling speed is constant), larger normal displacement loads $L_{Z}$ correspond to higher normal stresses. It is consistent with actual rolling conditions, as an increase in normal displacement loads $L_{Z}$ leads to an increase in rolling pressure, and the internal forces of the strip to resist the rolling pressure also increase. Therefore, normal stress increases with an increase in $L_{Z}$.

**Figure 19:**
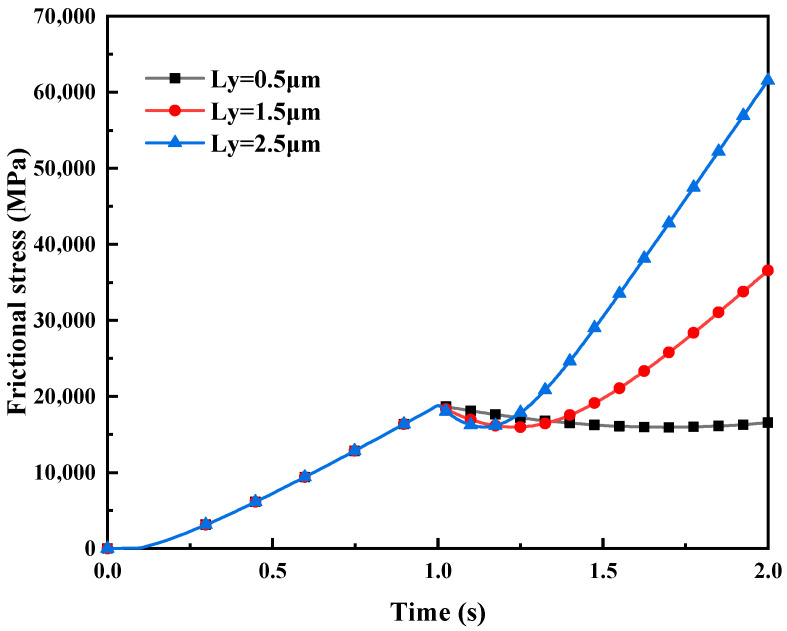
The impact of different displacement loads L_Y on friction stress in mixed lubrication with σ_2 = 5 μm.

Friction stress. Figure 19 illustrates the impact of different tangential displacement loads $L_{Y}$ on friction stress in mixed lubrication. As observed from Figure 19, the friction stress is non-zero during the normal loading process from 0 s to 1 s, which is due to the occurrence of the lateral contact of asperities during the indentation process, resulting in tangential stress. When the initial rolling pressure reaches a steady state at 1 s (i.e., the indentation depth $L_{Z}$ remains constant at 1 μm), a tangential displacement load $L_{Y}$ is applied. The simulation result reveals that the friction stress decreases with the increase of tangential displacement load $L_{Y}$ from 1 s to 1.2 s, and increases non-linearly with the growth of $L_{Y}$ from 1.2 s to 2 s. It is due to the gradual expansion of the lateral contact area of asperities resulting from the increase of the tangential displacement load in the initial stage of slip, which reduces the friction stress between contact asperities. However, with the accumulation of slip time, the friction stress enhances significantly, owing to the plowing effect produced by the severities of the roll and the strip during the slip contact, which improves the sliding resistance of the asperities on the strip surface. The resistance of the plowing effect is a component of the friction force. When the tangential displacement load $L_{Y}$ per unit time is larger (i.e., the rolling speed is faster), the plowing effect on the strip becomes more pronounced, and the resistance of the plowing effect becomes larger, increasing friction stress.

**Figure 20:**
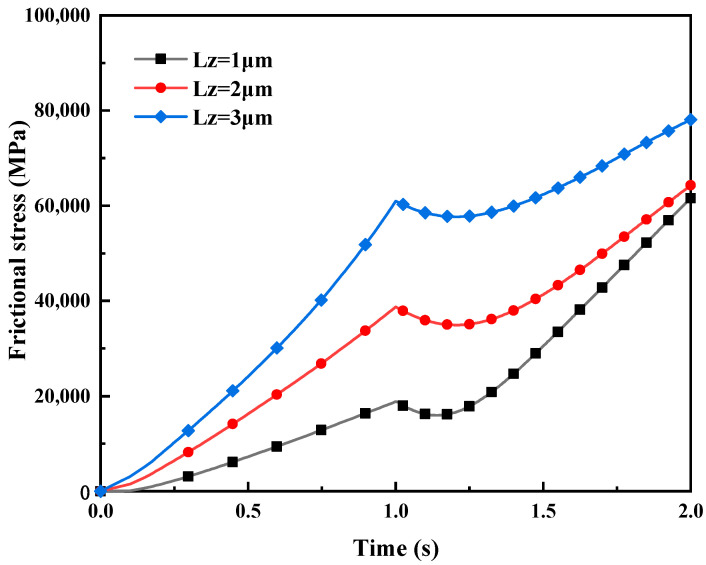
The impact of different displacement loads L_Z on friction stress in mixed lubrication with σ_2 = 5 μm.

Figure 20 depicts the influence of different normal displacement loads $L_{Z}$ on friction stress in mixed lubrication. The rolling pressure is linearly related to the rolling displacement and has a significant impact on the lubricant flow rate and viscosity at the rolling interface, which are important factors directly affecting friction resistance. Simulation results indicate that, when the tangential displacement load per unit time remains constant ($L_{Y}=2.5\;\mu m$) (i.e., the rolling speed is fixed), an increase in normal displacement load $L_{Z}$ causes a corresponding increase in rolling and normal pressure borne by the lubricating oil film. It increases in viscosity between the oil film molecular layers, leading to an increase in shear stress during sliding. Consequently, the friction stress also increases.

**Figure 21:**
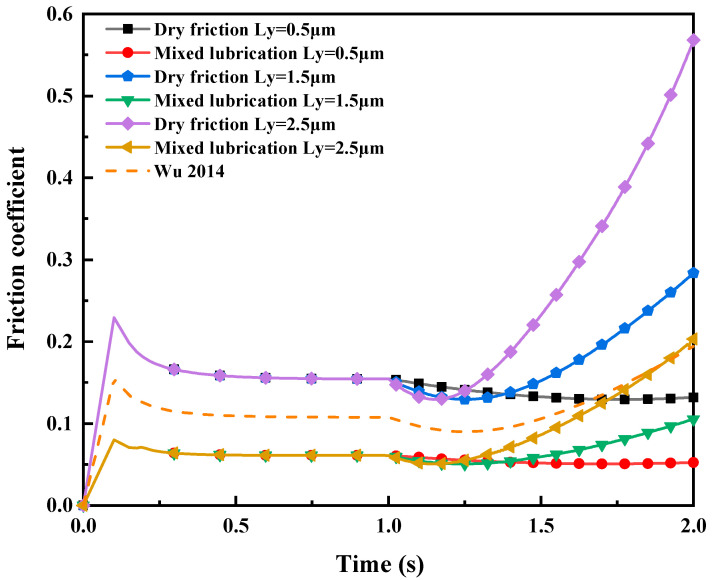
The influence of different tangential displacement loads L_Y on friction coefficient in two different working conditions with σ_2 = 5 μm, and compare with the data from literature [4].

Friction coefficient. Figure 21 illustrates the impact of different tangential displacement loads $L_{Y}$ on the friction coefficient in dry friction and mixed lubrication. The contact area of the rough asperities at the rolling interface is integrated using post-processing, and the corresponding normal pressure and friction force are obtained by combining Figure 17 and Figure 19. The friction coefficient variation is then computed by dividing the friction force by the normal pressure. The data-processing method of the dry friction model is consistent with the mixed lubrication model.

According to the simulation results, the friction coefficient is not zero during the normal loading from 0 s to 1 s owing to the tangential force generated by the lateral contact between the roller’s micro-asperities and the metal strip. From 1 s to 1.2 s, the friction coefficient decreases with the increase of tangential displacement load $L_{Y}$, because, in the initial tangential load application stage, the elastic deformation of the micro-convex bodies on the contact point of the roller is relatively low, while the metal strip at the contact point is in a plastic flow state, resulting in a slight increase in the contact area and a slight decrease in the friction coefficient. From 1.2 s to 2 s, the elastic deformation of the micro-asperities on the roller grows stronger with the accumulation of tangential displacement load $L_{Y}$. The adhesion and plowing effects of the rolling interface micro-asperities become more pronounced, resulting in an increase in friction stress and a decrease in normal stress. Therefore, the friction coefficient is increasing. The simulation results are consistent with the literature [4]. The literature estimated the total friction stress in the mixed-lubrication-by-contact-area ratio, and derives the interface friction coefficient via a post-processing calculation, which is comparable to the simulated simulation trend. During the normal loading and slip stages, the friction coefficient in mixed lubrication is always slightly lower than that of dry friction, because the lubricating oil involved in the rolling process fills the surface irregularities, reducing the direct contact of the micro-asperities and, thus, reducing the friction coefficient.

**Figure 22:**
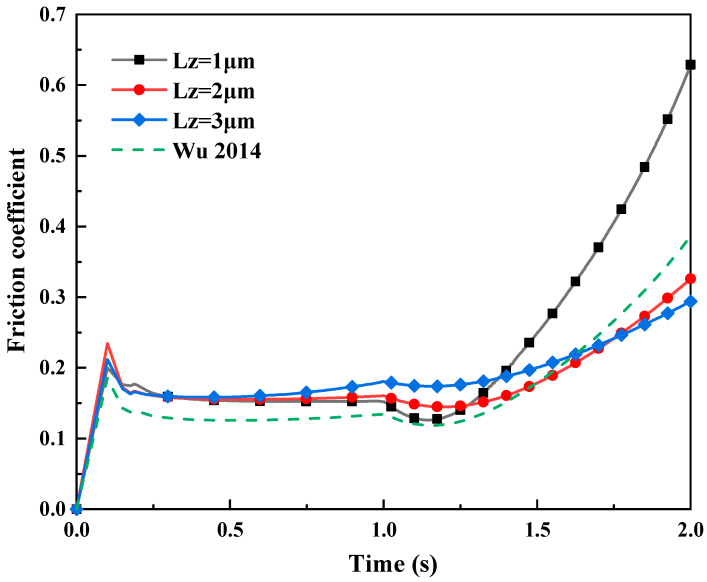
The influence of different normal displacement loads L_Z on friction coefficient in mixed lubrication with σ_2 = 5 μm, and compare with the data from literature [4].

Figure 22 presents the influence of different normal displacement loads $L_{Z}$ on the friction coefficient in mixed lubrication. The contact area of the asperities at the rolling interface was integrated through post-processing. Furthermore, the normal pressure and friction force corresponding to the normal stress and friction stress were calculated to determine the variation law of the friction coefficient by combining Figure 18 and Figure 20. And the variation law of the friction coefficient is similar to the literature [4], which verified the accuracy of the simulation. When the tangential displacement load $L_{Y}$ per unit time is constant ($L_{Y}=2.5\;\mu m$) (i.e., the input rolling speed is constant), the greater the normal displacement loads $L_{Z}$, the higher the contact pressure between the micro-asperities. Moreover, the friction coefficient decreases with an increase in contact pressure.

## 5. Conclusions

To reveal the contact characteristics of the rolling interface in the working zone under mixed lubrication, the roughness models for mixed lubrication were established based on Gaussian distribution theory, and the three-dimensional FSI simulation was carried out. Several meaningful conclusions were drawn as follows:

1. The lubricating oil film in the rolling interface plays a crucial role in reducing direct contact with micro-asperities, decreasing the friction, balancing the load, and suppressing uneven deformation of the strip. Therefore, when studying the rough contact conditions of the rolling interface at the micro scale, the effect of lubricating oil film cannot be ignored.
2. The simulation results of different roughness models show that different roughness and rolling conditions significantly affect the normal contact and tangential sliding behavior of rough surface micro-asperities. In the same conditions, the surface with a lower roughness has a more uniform distribution of wave peaks and valleys, and the number of rough peaks participating in contact is more significant; also, the bearing capacity of the lubricating oil film between the interfaces is improved.
3. When there is tangential sliding between the rolling mill and the strip, the normal stress decreases non-linearly with the sliding increase. In contrast, the friction stress first decreases and then rises rapidly with the increasing sliding. It is due to adhesion and plowing effects causing the elastic–plastic deformation of the contacting micro-asperities and forming plowing grooves on the strip’s surface during sliding, increasing the contact area and sliding resistance.
4. When the rolling speed between the rolling mill and the strip is constant, the normal and friction stresses created by the contact of the micro-asperities at the rolling interface grow as the indentation depth increases. Still, the friction coefficient reduces as the contact pressure increases. In mixed lubrication, the normal pressure borne by the lubricating oil film rises with the increasing normal displacement, resulting in a rise in viscosity between the oil film molecular layers, which increases the friction stress.

## Figures and Tables

**Table 1:** Performance parameters of materials.

Parameter	Value	Unit
Young’s modulus of roll 9Cr	200	GPa
Poisson’s ratio of roll 9Cr	0.3	_
Density of roll 9Cr	7850	$Kg\cdot m^{-3}$
Young’s modulus of strip 5052	71	GPa
Poisson’s ratio of strip 5052	0.3	_
Density of strip 5052	2770	$Kg\cdot m^{-3}$
Viscosity of lubricant N54	1.06 (25 °C)	$Kg\cdot m^{3}$
Density of lubricant N54	889	$Kg\cdot m^{-3}$

## Data Availability

Not applicable.
